# Long-read microbial genome assembly, gene prediction and functional annotation: a service of the MIRRI ERIC Italian node

**DOI:** 10.3389/fbinf.2025.1632189

**Published:** 2025-06-30

**Authors:** Sandro Gepiro Contaldo, Antonio d’Acierno, Lorenzo Bosio, Francesco Venice, Elisa Li Perottino, Janneth Estefania Hoyos Rea, Giovanna Cristina Varese, Francesca Cordero, Marco Beccuti

**Affiliations:** ^1^ Department of Computer Science, University of Torino, Turin, Italy; ^2^ Laboratorio InfoLife, Consorzio Interuniversitario Nazionale per l’Informatica, Roma, Italy; ^3^ Institute of Food Sciences, Consiglio Nazionale delle Ricerche-Istituto di Scienze dell’alimentazione, Avellino, Italy; ^4^ Department of Life Sciences and Systems Biology, Mycotheca Universitatis Taurinensis, University of Torino, Turin, Italy

**Keywords:** genome assembly, functional annotation, gene prediction, HPC and cloud service, reproducible analysis

## Abstract

**Background:**

Understanding the structure and function of microbial genomes is crucial for uncovering their ecological roles, evolutionary trajectories, and potential applications in health, biotechnology, agriculture, food production, and environmental science. However, genome reconstruction and annotation remain computationally demanding and technically complex.

**Results:**

We introduce a bioinformatics platform designed explicitly for long-read microbial sequencing data to address these challenges. Developed as a service of the Italian MIRRI ERIC node, the platform provides a comprehensive solution for analyzing both prokaryotic and eukaryotic genomes, from assembly to functional protein annotation. It integrates state-of-the-art tools (e.g., Canu, Flye, BRAKER3, Prokka, InterProScan) within a reproducible, scalable workflow built on the Common Workflow Language and accelerated through high-performance computing infrastructure. A user-friendly web interface ensures accessibility, even for non-specialists.

**Conclusion:**

Through case studies involving three environmentally and clinically significant microorganisms, we demonstrate the ability of the platform to produce reliable, biologically meaningful insights, positioning it as a valuable tool for routine genome analysis and advanced microbial research.

## 1 Introduction

Accurate reconstruction and functional annotation of microbial genomes are essential for understanding microorganisms’ biological roles, evolutionary dynamics, and biotechnological potential. The advent of long-read sequencing technologies has significantly enhanced the ability to generate high-quality genome assemblies.

However, transforming these raw sequencing data into meaningful biological insights requires advanced computational skills. Researchers must integrate various tools, develop reproducible workflows, and manage computing infrastructures, which can be challenging for those without a strong background in computer science.

To our knowledge, no user-friendly online workflow currently integrates all these tasks into a single working environment supporting prokaryotic and eukaryotic organisms. For instance, the Galaxy Europe (The Galaxy Community, 2024) community provides a rich library of bioinformatics tools, including long-read assemblers such as CANU (Koren et al., 2017) and Flye (Kolmogorov et al., 2019), as well as gene prediction and annotation tools like Prokka (Seemann, 2014) for prokaryotes and BRAKER3 (Bruna et al., 2021) for eukaryotes. Despite this, it lacks a workflow that integrates all these tools into a single, coherent pipeline applicable to both genomic domains. This creates a barrier to accessibility and reproducibility, particularly for researchers without advanced computational skills. Similar considerations apply to most workflows hosted on WorkflowHub (Gustafsson et al., 2024), where multiple pipelines exist, typically based on powerful workflow managers such as Nextflow (Di Tommaso et al., 2017), Snakemake (Köster and Rahmann, 2012), and Common Workflow Language (CWL) (Common workflow language documentation, 2025). However, these pipelines are often tailored to specific organisms or analysis goals and do not offer a unified design for complete automation. An exception to this is the CLAWS (CNAG’s Long-read Assembly Workflow in Snakemake) (Gomez-Garrido, 2024). This workflow supports long-read assembly using tools such as Flye (Kolmogorov et al., 2019) and NextDenovo (Hu et al., 2024), followed by polishing with Hypo (Kundu et al., 2019) and NextPolish (Hu et al., 2019), and evaluation with BUSCO (Manni et al., 2021b; Manni et al., 2021a). However, unlike our work, CLAWS does not utilize multiple assemblers to improve assembly quality and does not include any steps for gene prediction or functional annotation. Finally, the MGnify platform (Richardson et al., 2022), provided by EMBL-EBI, is conceptually similar to our proposed system; however, its focus differs. MGnify was developed explicitly for metagenome analysis, facilitating the assembly and annotation of genomics sequences of microbiomes found in several biomes. From the technical point of view, its workflows are described using CWL like ours; however, the web platform is built on the Django web framework.

Extracting biological insights from raw sequencing data derived from microbes in pure culture presents significant computational challenges within the SUS-MIRRI.IT project (http://www.sus-mirri.it), launched in 2022 with funding from the Italian government under the NextGenerationEU National Recovery and Resilience Plan (PNRR), we have developed a high-performance online service for microbial genome analysis, with a particular focus on tool integration, workflow reproducibility, and infrastructure management. This initiative aims to enhance the sustainability of MIRRI-IT, the Italian node of the pan-European Microbial Resource Research Infrastructure MIRRI ERIC (https://www.mirri.org), by consolidating microbial data from the Italian collections and providing open-access analysis tools for the research community. In detail, this service is part of the broader Italian Collaborative Working Environment (ItCWE - https://susmirri-mbrc.di.unito.it/), serving as the primary access point for SUS-MIRRI.IT services of the Italian MIRRI ERIC node. It represents a significant advancement toward a user-centered scalable bioinformatics service for microbial research. Indeed, the service provides comprehensive processing of long-read data, covering all steps from assembly to gene prediction and functional annotation for both prokaryotic and eukaryotic genomes. It combines user-friendliness with advanced computational capabilities and includes quality evaluation tools to guarantee reliable and reproducible analyses. Three key innovative aspects characterize our service. 1) *Ease of use*, an intuitive web application allows users to easily setup and execute their data analyses. Additionally, a dedicated post-processing web tool provides centralized access to enriched annotations and metadata by querying multiple external repositories, thereby facilitating biological interpretation and extracting meaningful insights from analysis outcomes. 2) *High-performance computing exploitation*, the service transparently leverages HPC infrastructure to accelerate the analysis, enabling the combination of outputs from multiple assembling tools (e.g., Canu (Koren et al., 2017), Flye (Kolmogorov et al., 2019), wtdbg2 (Ruan and Li, 2020)) to enhance the performance, completeness, and accuracy of genome assemblies. 3) *Easy reproducibility and evaluation of the analysis*, based on the Common Workflow Language (CWL) and containerized with Docker, the pipeline ensures complete transparency and portability. It integrates automated result evaluation using standard metrics, such as N50 and L50, and more advanced metrics like evolutionarily informed expectations of gene content from near-universal single-copy orthologs, supporting standardized quality assessment throughout every analysis phase.

To validate the utility of our service, we selected three microorganisms of clinical and environmental significance from the TUCC culture collections: *Scedosporium dehoogii* MUT6599 (Florio Furno et al., 2022), *Klebsiella pneumoniae* TUCC281, and *Candida auris* TUCC287.

## 2 Methods

The proposed service utilizes a modular architecture that operates on a hybrid computational infrastructure, integrating both cloud computing and High-Performance Computing (HPC).

The cloud system is built on the open-source OpenStack cloud computing infrastructure. It utilizes various resources from the open-access High-Performance Computing for Artificial Intelligence (HPC4AI) data centre (https://hpc4ai.unito.it) at the University of Turin, which includes over 2,400 physical cores, 60 TB of RAM, 120 GPUs (NVidia T4/V100/A40), 25 Gb/s networking, and four different storage classes with distinct characteristics.

The use of OpenStack enables the implementation of a robust Deployment-as-a-Service (DaaS) feature. This feature allows for the portable modeling of user-defined platforms (IaaS and PaaS) and automates their deployment on a virtualized infrastructure.

In contrast, the HPC subsystem is orchestrated by BookedSlurm, an extension of Slurm specifically designed to facilitate node booking through a user-friendly web calendar interface. It comprises 68 nodes of HPC4AI laboratory with Intel architecture (each with 36 cores and 128 GB of RAM), four nodes with Arm architecture (Ampere Altra, featuring 80 cores, 512 GB of RAM, 2 x A100 GPUs, 2 x BF2 DPUs, Ethernet 100 Gb/s), four additional Intel-based nodes (with 40 cores, 1 TB of RAM, T4 and V100 GPUs, Ethernet 56 Gb/s), and four nodes with ARM architecture (80 cores, featuring Grace Hopper Superchips). Furthermore, multiple storage systems use BeeGFS and LUSTRE, configured in an all-flash setup.

**FIGURE 1:**
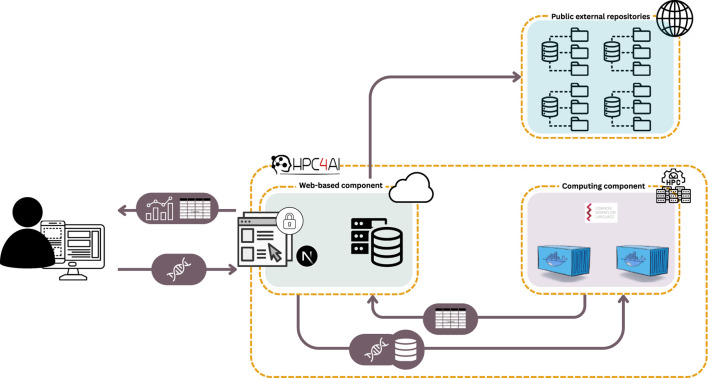
System architecture overview. Users upload data through the web-based component, which serves as the system’s interface in the cloud. The HPC-based computing component then processes the data, which employs Common Workflow Language and Docker containers to ensure reproducible and scalable analysis. Results are returned in tabular format and visualized through the web interface. The system also interacts with public external repositories to enrich analyses with additional metadata or reference data.

As illustrated in Figure 1, two primary service components are then instantiated on top of these two sub-infrastructures:

• The Computing Component manages the execution of the developed data analysis workflows on an HPC infrastructure, interacting with the web-based module to process user data according to predefined procedures. It receives raw data, processes it in parallel across multiple HPC nodes, and returns the results, which are then made available for visualization within the web-based component.
•The Web-based Component, operating on virtual machines within a cloud infrastructure, is responsible for data upload, configuration of analysis parameters, and result visualization. It features a user interface designed to ensure seamless interaction and an intuitive user experience, while also facilitating access to the underlying computational resources and serving as a central hub for connecting analysis outcomes with external repositories offering tools to support biological interpretation and the extraction of meaningful insights.

Another essential aspect of implementing the service is defining the data analysis workflow, which is intentionally not hard-coded into the computing component. Indeed, the developed workflow is provided as input to the computing component. This approach was chosen to facilitate the implementation of future services, which will primarily require redefining the workflow and associated web components with minimal impact on the computing component.

In the rest of this section, we focus on describing the considered analysis workflow and the service’s main components.

### 2.1 Data analysis workflow

The workflow described in this paper enables accurate genome assembly, gene prediction, and functional annotation of microbial organisms using long-read sequencing data. Its modular design supports both prokaryotic and eukaryotic genomes. The pipeline ensures high-quality, biologically meaningful results by integrating multiple cutting-edge tools and a systematic evaluation strategy. It offers a robust and scalable approach for microbial genome analysis, well-suited for complex or data-limited scenarios and ready for downstream comparative and functional studies.

**FIGURE 2:**
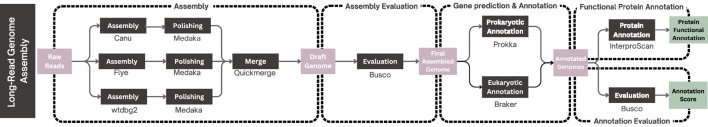
Overview of the long-read genome assembly workflow. The pipeline begins with assembling long-read sequencing data using three assemblers (Canu, Flye, wtdbg2), then polishing with Medaka and merging with Quickmerge. The best assembly is selected based on BUSCO and N50 scores. Gene prediction is performed using Prokka for prokaryotes and BRAKER for eukaryotes, followed by BUSCO-based annotation evaluation. Finally, functional annotation of predicted proteins is conducted using InterProScan.

Observe that the workflow is designed to be flexible, relying on parameter settings recommended by the developers of each integrated tool. Specifically, as the three assemblers included in the workflow are compatible with Nanopore, PacBio, and PacBio HiFi data, users can specify the sequencing technology used in their dataset through the graphical user interface (GUI). As sketched in Figure 2, it is structured into four main steps, each corresponding to a specific phase of the microbial genome analysis process: assembly phase, assembly evaluation phase, gene prediction and annotation phase and functional protein annotation phase.

#### 2.1.1 Assembly phase

The first phase of this workflow is dedicated to assembling long raw sequencing reads, a crucial step in reconstructing high-quality genomic sequences. Even if the current assembler produces reliable and high-quality results, our approach aims to refine and enhance genome assembly by integrating outputs from multiple assemblers. This strategy leverages the strengths of different assembly algorithms, which may yield better results depending on their specific ability to handle different genomic features and sequencing data characteristics.

To achieve this, we independently assembled the raw sequencing reads using three popular long-read assemblers: Canu, Flye, and wtdbg2. Then, the results of these assemblers are combined because these tools utilize different methods for managing sequencing errors and structural variations, providing complementary advantages. Each assembler employs distinct computational strategies to handle sequencing errors and structural variations, offering complementary advantages. Given the high computational demands of long-read assembly, these processes are executed in parallel within our computational infrastructure, enabling significant reductions in processing time. This parallelization ensures efficient resource utilization and facilitates the timely completion of large-scale sequencing projects, making it particularly beneficial for studies requiring rapid genome reconstruction.

Following the initial assembly phase, a polishing step is performed to correct sequencing errors and enhance base-level accuracy. For this, we employ Medaka (Oxford Nanopore Medaka, 2025), a deep-learning-based polishing tool designed explicitly for Oxford Nanopore sequencing data. Medaka applies a neural network model to refine the raw assemblies, correct miscalls, and improve sequence reliability, which is crucial for downstream applications such as gene annotation and variant detection.

Once the assemblies have been polished, they are refined through a merging process using Quickmerge (Chakraborty et al., 2016). This tool integrates pairwise assemblies by leveraging high-confidence alignments between contigs, effectively combining complementary structural information to enhance contiguity and completeness. By merging the outputs of multiple assemblers, we generate a more comprehensive and structurally accurate genome assembly, reducing fragmentation and improving the overall representation of complex genomic regions.

#### 2.1.2 Assembly evaluation phase

After obtaining the set of assemblies from the previous step, we aim to systematically identify and retain the highest-quality assembly for each sample. To achieve this, we evaluate all merged assemblies using BUSCO (Manni et al., 2021b; Manni et al., 2021a), a widely used tool for assessing genome completeness and redundancy based on the presence of conserved orthologous genes. This metric provides a biologically meaningful measure of assembly quality by estimating the proportion of expected single-copy orthologs that are complete, fragmented, or missing.

In evaluating assemblies, we incorporate completeness and contiguity metrics, specifically the BUSCO score and the N50 value. The BUSCO score is related to the presence in the generated assembly of orthologous genes from closely related organisms that are considered essential to sustain cellular activities, while the N50 measures assembly contiguity.

We follow a flexible selection process for each sample to identify the optimal assembly. Users can prioritize the BUSCO score or the N50 value based on their needs. Assemblies are first ranked according to the chosen primary criterion. In cases where multiple assemblies have identical values for the selected metric, the secondary criterion is used to break the tie. We retain the first assembly if a tie persists after considering both metrics. This approach ensures consistency while allowing users to tailor the selection process to their project goals.

Using this hierarchical selection strategy, we guarantee that the selected assemblies are the most biologically complete, contiguous, and high-quality versions available for each sample. This method improves the reliability of subsequent analyses, such as functional annotation, comparative genomics, and evolutionary studies.

#### 2.1.3 Gene prediction and annotation phase

Gene prediction and annotation are crucial steps in analyzing microbial genomes. In our workflow, we approach this process in two distinct ways, depending on whether the microorganism is prokaryotic or eukaryotic. Each approach utilizes specialized tools and strategies tailored to the unique characteristics of the genomes within each domain, ensuring accurate and comprehensive annotation results. For prokaryotic genomes, we utilize Prokka (Seemann, 2014) to ensure that the predicted genes are accurate in location and classified adequately according to their biological function. Prokka integrates several state-of-the-art, *de novo* gene prediction algorithms, providing a high-confidence set of predicted genes. In addition to genes, Prokka also identifies other key genomic features, such as ribosomal RNA (rRNA) genes and transfer RNA (tRNA) genes. The tool also performs functional annotation by matching predicted genes to external databases, such as NCBI and UniProt, allowing functional roles to be assigned to the identified genes based on sequence homology. This ensures that the predicted genes are accurate in location and classified adequately according to their biological function.

For eukaryotic genomes, annotation is carried out using Braker3 (Bruna et al., 2021), an advanced gene prediction tool built around the AUGUSTUS gene predictor (Keller et al., 2011). Braker3 incorporates external evidence to refine gene models, resulting in highly accurate gene predictions. It is essential to highlight that without RNA-seq data, BRAKER3 uses a protein-based approach, mapping protein sequences from closely related species to the target genome. These proteins are used as extrinsic evidence, and cross-species mapping of homologous proteins helps to infer the coordinates of exons, improving prediction accuracy.

A key feature of our approach is the incorporation of curated, phylum-specific protein datasets from UniProt (https://www.uniprot.org/). These datasets provide valuable additional evidence for gene prediction, particularly for the supported fungal phyla, including Ascomycetes, Basidiomycetes, Mucoromycetes, and Oomycetes. These curated datasets allow us to fine-tune gene models by incorporating additional biological context specific to the phyla being studied.

The prokaryotic and eukaryotic gene prediction approaches used in our pipeline are based on an *ab initio* strategy, which relies on intrinsic genomic signals such as start and stop codons, as well as other regulatory elements, to predict genes. While these methods do not require prior knowledge of gene structures, they are complemented by external evidence in the case of eukaryotic genomes, where protein homology mapping is used to improve prediction accuracy.

The final output from this gene prediction and annotation phase is an annotated set of genes, complete with genomic coordinates and predicted protein products. This comprehensive gene annotation is the foundational dataset for downstream analyses, including functional characterization and metabolic pathway reconstruction.

#### 2.1.4 Annotation evaluation phase

As we did in the assembly evaluation phase, we want to evaluate the results of the previous step using BUSCO (Manni et al., 2021b; Manni et al., 2021a). In this case, however, we are evaluating the annotated genomes to assess the completeness and accuracy of the annotations, with a specific focus on the predicted protein-coding sequences.

We use the BUSCO score, specifically on protein-coding sequences, to assess the completeness and accuracy of the annotations. This step provides crucial insights into the quality of the genome annotation, ensuring that it aligns with the protein content for the organism and/or its taxonomic group. By evaluating the presence and completeness of conserved single-copy orthologs, we can assess how well the predicted proteins align with the expected protein set for the organism.

This step ensures the reliability and completeness of the genome annotations, laying the foundation for subsequent analyses and interpretations.

#### 2.1.5 Functional protein annotation phase

To gain deeper insights into the functional characteristics of the predicted protein sequences, we utilize InterProScan (Jones et al., 2014; Blum et al., 2020), a widely used tool that integrates multiple functional annotation databases. Using amino acid sequences predicted by Prokka or BRAKER3, we perform a comprehensive analysis to identify protein domains, conserved motifs, and protein families. This approach allows us to infer potential biological functions associated with the sequences by leveraging well-established databases such as Pfam (Mistry et al., 2021), Panther (Thomas et al., 2003), Superfamily (Gough and Chothia, 2002), among others.

Molecular functions can be predicted by estimating the degree of homology between the predicted proteins and these curated resources. Interpro annotations also contain information that communicates with databases such as Gene Ontology (https://geneontology.org/) and REACTOME (https://reactome.org/), allowing for predicting whether a protein belongs to specific metabolic pathways or reactions.

Furthermore, integrating multiple databases enhances the robustness of our annotations, providing a more comprehensive functional characterization of the protein-coding genes identified in our dataset.

### 2.2 Computing component

This component is directly tied to the HPC infrastructure and leverages the custom BookedSlurm API to manage and execute compute-intensive tasks. The system enables parallel execution of multiple analysis workflows, improving both resource utilization and system scalability. In particular, the component is implemented to support the execution of workflows specified through the Common Workflow Language (CWL) (Common workflow language documentation, 2025), an open standard designed for defining and linking command-line tools to build portable and reproducible workflows. Additionally, every tool utilized in the workflow is encapsulated within a Docker image (Docker official site, 2025). Observe that we chose to use CWL for workflow development due to several key advantages: 1) CWL is a declarative coordination language, which, while generally less expressive than imperative alternatives (such as Snakemake or Nextflow), facilitates the application of rewriting techniques for optimization by Workflow Management Systems, thereby enhancing performance portability; 2) CWL offers portability and scalability across diverse software and deployment environments; and 3) our project serves as a case study for the CWL community, as highlighted in the CWL gallery (https://www.commonwl.org/gallery).

Currently, the service is free for all users involved in the SUS.MIRRI.IT project and those affiliated with the Italian node of MIRRI-ERIC. For other users, we are evaluating a credit-based system in which tokens can be purchased to access and run the workflows. This policy is being considered to ensure the long-term sustainability of the system.

### 2.3 Web-based component

This component is hosted on the Cloud sub-infrastructure and provides a user-friendly web application built using state-of-the-art tools and frameworks.

The front-end of the application was implemented using Next.js, a popular framework built on React that supports both client- and server-side rendering, enabling performance optimization. We integrated a self-hosted MinIO (Minio official site, 2025) instance to provide efficient object storage capabilities for temporary data storage. Within MinIO, a central bucket is configured to store user-specific data; for each user, a dedicated directory is created inside this bucket, containing separate subfolders for input and output files. User authentication is managed via Keycloak, which has been deployed with a dedicated realm to isolate the client and enable secure user authentication through a username-password mechanism. A PostgreSQL database has been set up to store metadata associated with each analysis, allowing users to access a history of their previously executed tasks. The resulting web application enables authenticated users to upload their data, execute analyses through the workflow, and download the corresponding output files. The system is publicly accessible at https://susmirri-workflow.di.unito.it.

**FIGURE 3:**
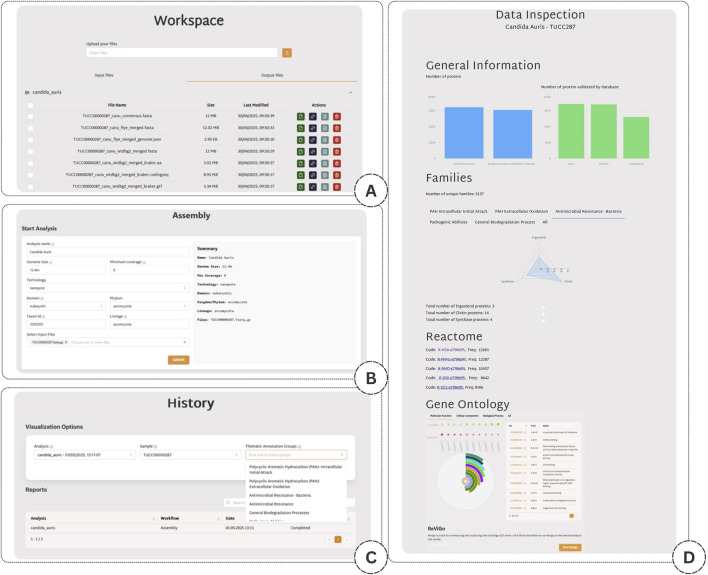
Overview of the application interface. **(A)** A file upload interface allowing users to manage their data. **(B)** The workflow configuration panel is used to launch new analyses and input metadata. **(C)** The analysis history window provides access to previously executed tasks. **(D)** The visualization dashboard displays results across four modules: General Information, Families, Reactome, and GO.

The application consists of four main windows, as illustrated in Figure 3. The first window (A) allows the user to upload a file into a user-specific file system. The second window (B) describes the analysis workflow, where the user can initiate a new analysis task by selecting the input file and specifying the associated metadata. The third window (C) displays the user’s analysis history, offering an overview of past tasks. From this window, the user can access the fourth window, which provides a visualization of the analysis results organized into the following four main blocks: General Information, Families, Reactome, and GO (D).

The General Information block contains a detailed summary of the downloadable results. This summary includes evaluating the genome assembly quality, as outlined in the Assembly Evaluation Phase. It also specifies the total number of identified amino acid sequences within the sample, alongside the number of these sequences validated as proteins by InterPro. Furthermore, the visualization displays the number of proteins validated by each specific InterPro database, including Pfam, Panther, and others.

In the Families block, users can explore the ecological lifestyles and biochemical capabilities of the analyzed species, providing critical insights into their potential applications across diverse biological and industrial contexts. Specifically, the users can explore protein families identified from predicted sequences using two complementary views. The first allows users to select four thematic annotation groups accessible via dedicated, interactive tabs. The relevant protein families are displayed in a radial plot to improve result interpretation, ordered by the number of annotated proteins. In contrast, the second view will enable users to directly inspect all identified families to which at least one protein has been assigned. This facilitates a detailed search for specific terms or functions of interest.

In the GO block, the Gene Ontology (GO) terms related to the sample are reported through a post-processing step that quantifies and assesses the significance of these terms. This step identifies overrepresented functional categories and provides insights into the biological roles potentially enriched in the dataset. In detail, for each GO term assigned to at least one protein in the sample, we computed an enriched p-value to assess whether its presence was more significant than expected by chance. To achieve this, we constructed a 2 × 2 contingency table and applied Fisher’s exact test, using the ‘greater’ alternative hypothesis to test for overrepresentation of the GO term within the sample. To populate the contingency matrix, we consider in the first row, associated to the numbers in the set of interest, in the first column the number of proteins in the sample annotated with the GO term of interest and in the second column the number of proteins in the sample not annotated with that GO term. The second row describes the background dataset, as the number of proteins annotated with the GO term in the reference background (excluding those already in the sample), in the first column, and the number of background proteins not annotated with the GO term, in the second column. Then, to accurately define the background frequency of each GO term, we queried the QuickGO API to retrieve the number of proteins annotated with the specific GO term, filtered by the relevant NCBI Taxonomy ID corresponding to the organism of interest. The total number of proteins annotated with any GO term within the same taxon was also retrieved to serve as the background population. This approach allowed us to compute an enriched p-value for each GO term observed in the sample, identifying significantly overrepresented terms statistically robustly. The GO results section, the top ten enriched GO terms are shown in both tabular format, showing the corresponding adjusted p-values, and through a radial plot that displays the number of annotated proteins for each GO term. Specifically, the central circle corresponds to the GO term with the highest number of annotated proteins. All other segments are color-coded proportionally, using the protein count of the central circle as the reference maximum. To further support interpretation, we include two bubble plots illustrating two key metrics for the top 10 GO terms: the adjusted p-values and the frequency of annotated proteins.

Moreover, to summarize and visualize long lists of GO terms, we integrate the REVIGO tool in this block. This connection allows users to automatically initiate a REVIGO job directly from our platform using the GO terms found in the sample. Once submitted, REVIGO processes the input to eliminate redundant or semantically similar terms. Then, it generates a semantic similarity-based graph that visually clusters related GO terms, helping researchers better interpret large-scale functional annotation data. The resulting graph provides an intuitive overview of the underlying biological processes, facilitating the identification of key themes and functional relationships within the dataset. This integration streamlines the analysis workflow by avoiding manual uploads and enables seamless visualization of GO term relationships.

Finally, in the Reactome block, the most frequently involved Reactome metabolic pathways are displayed at the bottom of the page. Each listed pathway includes a direct link to its corresponding page on the official Reactome website, where users can explore comprehensive details, including associated genes, reactions, and biological significance.

## 3 Results

In this section, we demonstrate the main features of our service, which include: 1) ease of use, through a user-friendly web interface and centralized access to enriched annotations; 2) integration with high-performance computing, enabling fast and accurate genome assembly by combining multiple tools; and 3) reproducibility and standardized evaluation, ensured by CWL-based, containerized workflows and automated quality assessment using both standard and advanced genomic metrics.

This will be carried out first by providing a detailed overview of the visualization windows introduced in the previous section, and secondly through a case study in which we selected three clinically and environmentally relevant microorganisms from the TUCC culture collections: Scedosporium dehoogii MUT6599 (Florio Furno et al., 2022), Klebsiella pneumoniae TUCC281, and Candida auris TUCC287.

Each experiment was executed on a single node of the Broadwell partition (i.e., with 2 Intel(R) Xeon(R) CPU E5-2697 v4 16-core processors, 128 GB RAM, and an OPA of 100 GB/s) to simulate standard user access to resources. In Table 1, we reported the total number of reads and the global execution time for each experiment.

**TABLE 1:** Reads number and runtime for three microbial species.

	Reads number	Runtime
*Candida auris*	846,401	6 h 3 min
*Klebsiella pneumoniae*	354,363	3 h 20 min
*Scedosporium dehoogii*	1 519 469	14 h 44 min

### 3.1 Case study: the functional analysis of three microorganisms

**FIGURE 4:**
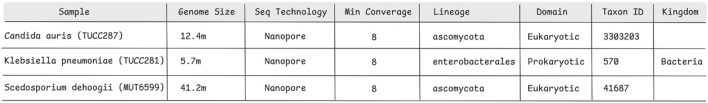
List of input parameters for each microorganism analysed.

The analysis starts by uploading the sequencing FASTQ file and specifying only a few parameters, such as genome estimated size, sequencing technology, minimum coverage, lineage, kingdom, domain, taxon ID, and the quality evaluation metric, without needing to handle the complex computational aspects associated with the use of HPC. These input parameters for each microorganism analysed are reported in Figure 4.

**FIGURE 5:**
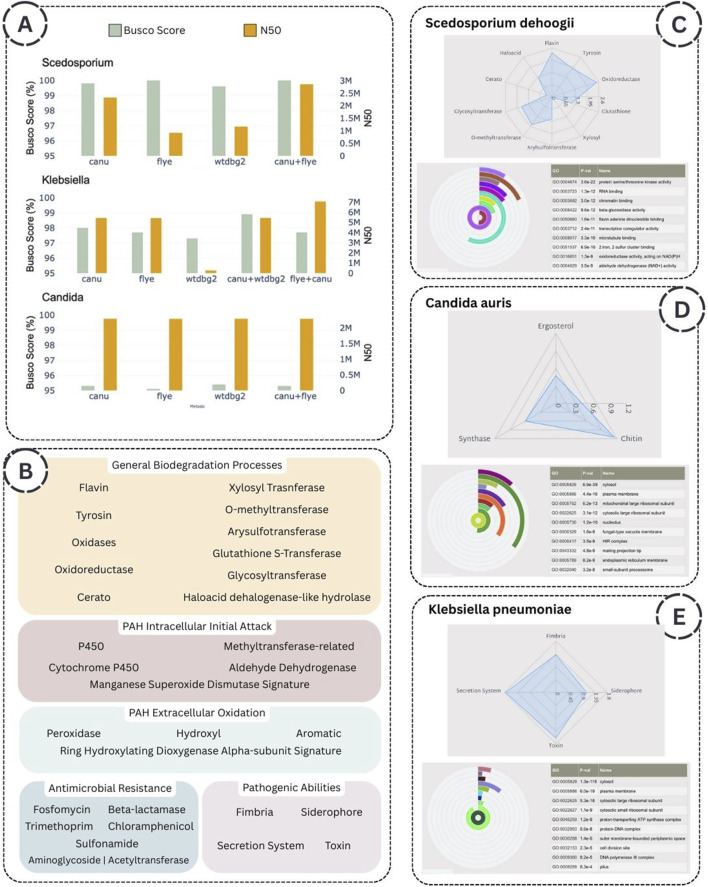
**(A)** Performance of the assembly algorithms, expressed by BUSCO scores and N50 values. **(B)** List of keywords associated with the five functional annotation groups. **(C)**, **(D)**, **(E)** Summary of key findings for *Scedosporium dehoogii*, *Candida auris*, and *Klebsiella pneumoniae*, respectively, including relevant protein families identified through radar plots and the top 10 most enriched Gene Ontology terms.

Two primary metrics are implemented for evaluating genome assemblies: the BUSCO score, which is recommended when focusing on gene prediction and functional annotation, and N50 values when the interests of the user are related to genome architecture, chromosomal organization, and evolutionary dynamics. In this context, we observed that the performance of genome assembly is enhanced through a couple of assembly algorithms, which benefit significantly from the computational power of HPC infrastructure. Specifically, for each species analyzed, the couple Canu and Flye assemblers outperform individual assemblers regarding N50 value (Figure 5A).

To give the possibility to the user to easily inspect the function of the microorganisms analyzed the four thematic annotation groups are: 1) Polycyclic Aromatic Hydrocarbon (PAH): Intracellular Initial Attack, 2) Polycyclic Aromatic Hydrocarbon (PAH): Extracellular Oxidation, 3) Antimicrobial Resistance, 4) General Biodegradation Processes, 5) *Pathogenic Abilities*. The keywords selected in each group are reported in Figure 5B.

*Scedosporium dehoogii* MUT6599 (Florio Furno et al., 2022) is a filamentous fungus isolated from the Livorno harbour (Mediterranean Sea, Italy), a site with a remarkable concentration of several pollutants, including Polycyclic Aromatic Hydrocarbons (PAHs), heavy metals, and microplastics. As the biodegradation potential of fungi isolated from highly polluted environments can be hypothesized based on genomic data (Venice et al., 2020), we aimed to characterize specific enzyme classes to evaluate a potential application in mycoremediation of this strain. Our analysis reveals a metabolic specialization towards the extracellular oxidation of organic pollutants, a pathway strongly associated with environmental biodegradation. These fungi exhibit an enriched repertoire of transferase enzymes, facilitating the conjugation of hydroxylated intermediates into glucosides, glucuronides, xylosides, sulfates, and methylated derivatives—a conserved detoxification mechanism among fungal species. Moreover, the functional enrichment analysis based on Gene Ontology revealed that among the top ten most enriched molecular function terms there are beta-glucosidase activity and dehydrogenase (NAD+) activity, which are critically involved in fungal-mediated metal detoxification processes (Priyadarshini et al., 2024). The radar plot reporting the General Biodegradation Processes annotation group and the radial bar chart showing the Molecular Functions term are reported in Figure 5C. All collected results are available for exploration at the following link https://susmirri-workflow.di.unito.it/examples/scedosporium_dehoogii.

*Candida auris* TUCC287 was isolated from a tracheal aspirate, and belongs to a species of multidrug-resistant yeasts that has rapidly spread globally, posing serious diagnostic and therapeutic challenges (Chowdhary et al., 2017). These fungal pathogens show high resistance rates—up to 90% to fluconazole and 30% to amphotericin B—with growing reports of resistance to echinocandins, the current first-line treatment for C. auris infections (Mishra et al., 2023). Accordingly, the workflow is designed to rapidly identify resistance genes, as evidenced by the number of proteins associated with antimicrobial resistance mechanisms in *Candida* species. This facilitates their accessibility for downstream comparative analyses. In detail, three terms are searched: *ergosterol*, *chitin*, and *1,3-beta-glucan synthase* as reported in the radar plot Figure 5D. Specifically, the ergosterol biosynthetic pathway enzymes are essential targets of several antifungals used to treat *Candida* (Dominique et al., 2003). Moreover, Echinocandins, which inhibit β (1,3)-glucan synthesis in the fungal cell wall, are effective against *Candida*. Sub-inhibitory exposure can induce compensatory chitin upregulation and increased cell wall chitin content (Louise et al., 2013). Observing the radial bar plot to report the first 10 more enriched Cellular Component terms (Figure 5D), it is possible to identify the HIR histone, and its pivotal role in modulating virulence of the human fungal pathogen was already reported in the *Candida* genus (Jenull et al., 2021). The complete set of results can be accessed and browsed through the following link https://susmirri-workflow.di.unito.it/examples/candida_auris.

*Klebsiella pneumoniae* is a well-known Gram-negative pathogen associated with multidrug resistance and nosocomial infections, requiring detailed genomic scrutiny to track resistance determinants and virulence factors (Wyres and Holt, 2016). We chose *K. pneumoniae* TUCC281, also isolated from a tracheal aspirate, as a benchmark for evaluating bacterial pathogenic strain. Also in this case, the workflow considers traits such as multidrug resistance to assess the pathogenicity of the selected strain. All findings from the analysis are made accessible for interactive browsing via the link below https://susmirri-workflow.di.unito.it/examples/klebsiella_pneumoniae, in which it is possible to explore the elevated number of families involved in the *Antimicrobial Resistance* annotation group. In Figure 5E, the radar plot reported the four keywords associated with *Pathogenic Abilities*, as well as secretion protein that is the first factor of virulence, the siderophore molecules, along with iron acquisition systems, play a critical role in bacterial pathogenicity, as several studies have demonstrated that their defective production or function is associated with a marked reduction in virulence (Behnoush et al., 2021). Fimbrial proteins, or pili, are filamentous surface structures of bacteria that play essential roles in adhesion to host tissues, colonization of surfaces, and bacterial motility. Notably, the terms *pilus* and *pilus organization* appear among the top 10 most enriched Cellular Component terms (Figure 5E) and Biological Process terms (available at the link above), underscoring the bacteria’s potential for host cell attachment.

## 4 Discussion

To facilitate the analysis of sequencing data from eukaryotic and prokaryotic genomes, we developed a computational framework designed to ensure reproducibility, accuracy, and user accessibility through a responsive and user-friendly interface for visualizing results. The system is tailored to meet the needs of computational experts specialized in microbiome research and experimental microbiologists seeking a clear and comprehensive overview of genomic data.

The system seamlessly integrates advanced computational tools with a user-friendly web interface, ensuring accessibility for users with varying levels of bioinformatics expertise. Moreover, the workflow is containerized and constructed using the CWL, enhancing reproducibility and portability. Specifically, the workflow consists of an initial phase dedicated to genome assembly from long-read sequencing data, employing three different algorithms: Canu, Flye, and wtdbg2. This is followed by a polishing step using Medaka and a merging phase using Quickmerge. The optimal assembly, regarding contig length and accuracy, is selected based on BUSCO and N50 scores. BUSCO score and N50 are key metrics to ensure a robust assembly and annotation output assessment. The workflow automatically selects the optimal final assembly based on these two indicators by combining the results of multiple state-of-the-art assemblers. Once the genome is assembled, gene prediction is performed using Prokka for prokaryotes and BRAKER for eukaryotes, followed by further quality assessment through BUSCO.

We incorporated a functional annotation step based on protein prediction using InterProScan to complement these analyses. To make the results accessible to domain experts and users less familiar with genomic data, we developed a dashboard reorganizing and visualizing the derived information. Specifically, we created semantic dictionaries focused on major molecular functions of applied interest, such as antimicrobial and antifungal resistance, and plastic degradation capability. These dictionaries identify proteins directly associated with the specified functions, which are visualized through radar plots. The dictionaries can operate at both the protein family and domain levels, and can be customized by users as needed.

Additionally, we explored the functional significance of the annotations by analyzing associated Gene Ontology (GO) terms. Beyond simple visualization, the framework enables users to compute the enrichment of GO terms both within the studied species and relative to higher taxonomic levels. Considering the hypergeometric distribution underlying gene-term associations, Fisher’s exact test is applied to calculate enrichment probabilities, selecting appropriate interest categories. GO terms can also be visualized within a semantic space using the Revigo tool. Finally, network-based functional analyses are provided through integration with Reactome, allowing users to explore pathway-level interactions.

The infrastructure offers a reliable and scalable microbial genome analysis solution in research and applied contexts, as illustrated by a case study involving three clinically and environmentally relevant microbial genomes. This framework offers a comprehensive and efficient tool for exploring a microorganism’s functional potential. The workflow can be applied to a wide range of microorganisms, and the customizable semantic dictionaries allow for an effective and accurate inspection of domains of interest.

## 5 Conclusion

In this study, we present a novel online computational service developed at the Italian node of the pan-European Microbial Resource Research Infrastructure (MIRRI-ERIC). To our knowledge, no comparable platform currently exists that enables the comprehensive processing of long-read sequencing data—from genome assembly and polishing to gene prediction and functional annotation—through an accessible web interface. This service facilitates the exploration of genomic features in microbial organisms via a structured and interactive visualization framework, supporting in-depth result inspection and fostering new insights into microbial functions.

## Data Availability

The raw data supporting the conclusions of this article will be made available by the authors, without undue reservation.
